# Donor-Site Complication of Severe Valgus Ankle Deformity in an Adult With Ehlers-Danlos Syndrome Following Free Vascularized Fibular Grafting

**DOI:** 10.7759/cureus.42397

**Published:** 2023-07-24

**Authors:** Melissa L Lipari, Steven E Laxson

**Affiliations:** 1 Podiatry, Legacy Health, Portland, USA; 2 Podiatry, Kaiser Permanente, Portland, USA

**Keywords:** ankle joint arthrodesis, syndesmotic screws, joint hypermobility syndrome, ehlers danlos syndrome, ankle valgus deformity, free fibula graft, free vascularized graft

## Abstract

Valgus deformity of the ankle joint is a well-known and relatively common donor-site complication of free vascularized fibular graft harvest in children. Due to children having naturally greater ligamentous laxity than adults, the tibiofibular syndesmosis can be compromised with the loss of the fibular shaft, leading to valgus ankle deformity (VAD). Syndesmotic stabilization with screws is commonly recommended in subsets of pediatric patients at the greatest risk of this complication. In adults, the occurrence of VAD is seldom reported in the literature following fibular graft harvest. As such, no recommendation for syndesmotic stabilization exists in the adult population. We present a case of end-stage VAD in an adult patient with Ehlers-Danlos syndrome (EDS) following free vascularized fibular graft harvest. We hypothesize that other patients with generalized joint hypermobility may face the same complication and, thus, recommend the consideration of syndesmotic stabilization or primary syndesmotic fusion at the time of graft harvest in this patient population.

## Introduction

Free vascularized fibular grafting (FVFG) is an attractive option for the treatment of large segmental bone defects and is the workhorse for orofacial reconstruction following tumor resection. The free fibular graft provides structural support, a tricortical contour for receiving dental implants, ease of harvest, copious length for various reconstruction options, and a long high-caliber vascular pedicle for microvascular anastomosis. Studies have also reported a relatively low donor site morbidity [1].

There is a scarcity of literature reporting on the donor-site complication of valgus ankle deformity (VAD) in the adult population following FVFG. This complication, however, is common in the pediatric population, with an incidence as high as 40% in some studies [2]. An explanation for this discrepancy lies in the greater prevalence of generalized joint hypermobility due to ligamentous laxity in children versus adults. A study of over 1,100 children aged four to seven years old revealed 64.6% had a Beighton score greater than four, indicating joint hypermobility [3]. Adults with this same pathology are often clinically categorized under the umbrella of Ehlers-Danlos Syndrome (EDS), which has numerous subtypes depending on the genetic mutation affecting collagen [4]. The overall prevalence of EDS is one in 5000 [5].

The occurrence of VAD following FVFG in children, although not fully understood, is likely multifactorial. It is partially mediated through ligamentous laxity causing proximal fibular migration. The deformity is then amplified by lateral tibial physeal atrophy due to increased pressures, causing relative overproduction of the medial versus the lateral tibial physis [2]. Another theory suggests that fibular artery transection during graft harvesting may result in premature closure of the distal fibular physis, reducing distal fibular growth. [2]. To mitigate this complication, it is generally recommended to preserve the distal 6 cm of the fibula [6]. In addition, fibular reconstruction, such as tibial strut or syndesmotic screw placement, may help prevent the development of VAD [7].

In the adult population, reported long-term donor site complications include leg weakness, ankle instability, great toe contracture, and decreased ankle mobility, but studies do not report the occurrence of VAD as a complication. One study of 157 adults following FVFG reported that despite the occurrence of the above complications, they saw no long-term functional limitations regarding the donor site [8]. We report on a case of a 57-year-old female with EDS who developed severe VAD following FVFG.

## Case presentation

**Figure 1:**
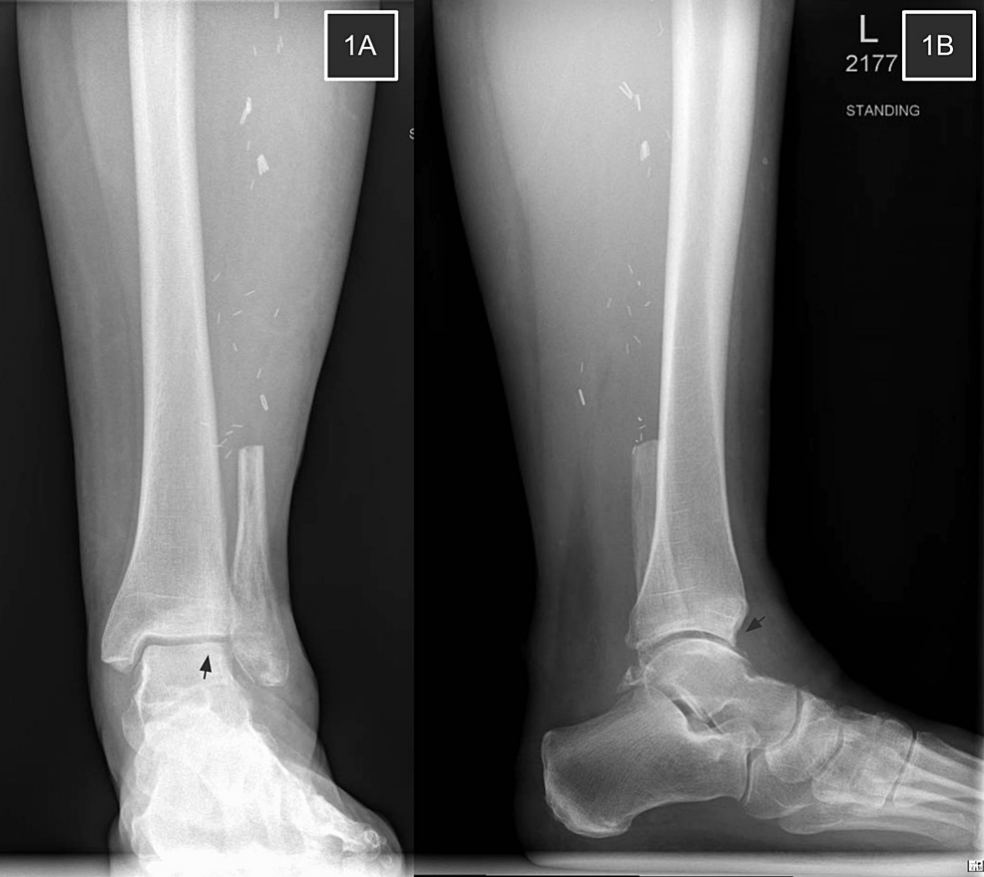
Ankle radiographs four months post-FVFG 1A shows irregularity of the lateral shoulder of the talar dome without valgus deformity of the ankle joint. 1B reveals a small anterior distal tibial osteophyte without talar subluxation in the sagittal plane.

**Figure 2:**
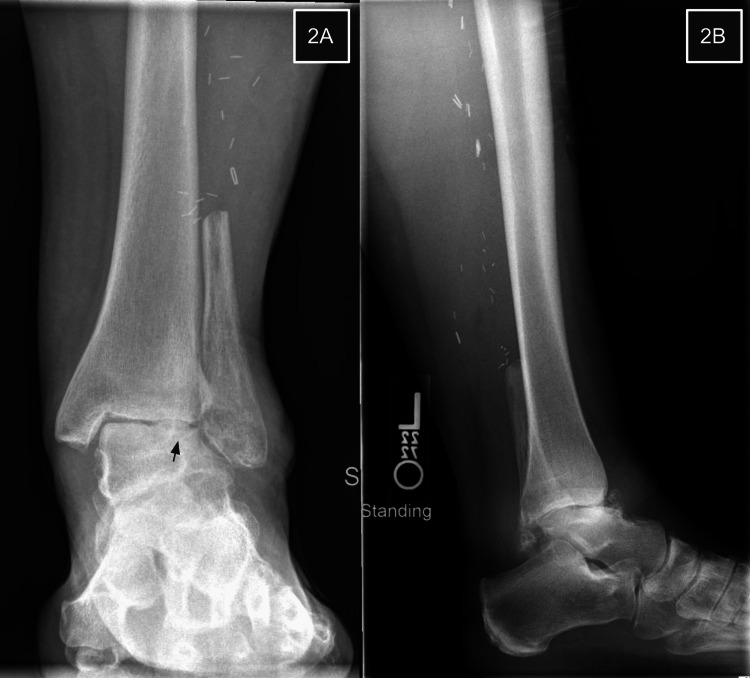
Ankle radiographs nine months post-FVFG 2A demonstrates interval collapse of the talus with valgus tilt and narrowing of the lateral tibiotalar joint and lateral gutter. Proximal displacement of the fibula is noted. The lateral projection (2B) reveals anterior displacement of the talus at the ankle joint.

This is a report of a 57-year-old female initially complaining of distal radioulnar joint instability with frequent dislocations who underwent an ulnar shortening osteotomy for treatment. She developed a nonunion of her ulnar osteotomy, which was revised with the assistance of the plastic surgery department with an FVFG from her left leg. Over 7 cm of the fibula was maintained for ankle stability. The patient was allowed to bear weight one week postoperatively and first experienced ankle pain and symptoms of instability at that time. X-rays were taken at four months postoperatively which did not show evidence of VAD (Figures 1A-1B). The patient unfortunately continued to have progressive ankle pain and instability despite physical therapy and bracing. Repeat ankle radiographs nine months post-FVFG demonstrated interval valgus tibiotalar joint collapse with proximal fibular migration (Figures 2A-2B). Her valgus ankle collapse progressed over the following months, as did her pain and disability.

She presented to the orthopedic department for evaluation. On physical exam, she had significant but reducible hindfoot valgus and mild reducible forefoot varus. She had significant pain and crepitation with ankle range of motion. The subtalar motion was preserved. She exhausted conservative treatment measures and elected for surgical intervention. Nonunion labs were ordered as well as an endocrine referral placed in the setting of multiple nonunions, including partial ulnar nonunion of the proximal graft site. This workup was negative, except for calcium deficiency, which was treated. In the interim, the patient was also diagnosed with EDS with a Beighton score of greater than five.

**Figure 3:**
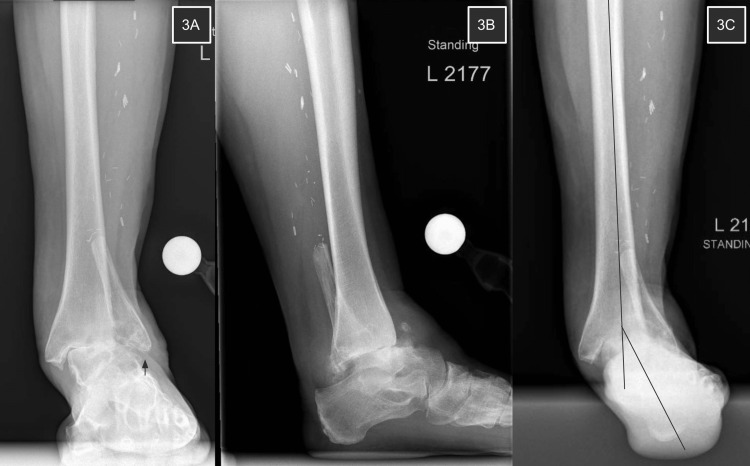
Ankle radiographs 20 months post-FVFG 3A shows severe tibiotalar joint valgus deformity of 15 degrees. The lateral malleolus has migrated proximally with its tip now near the level of the ankle joint. 3B demonstrates continued anterior talar subluxation and flattening of the talar dome. 3C is a hindfoot alignment view illustrating the patient’s severe hindfoot valgus secondary to her VAD.

**Figure 4:**
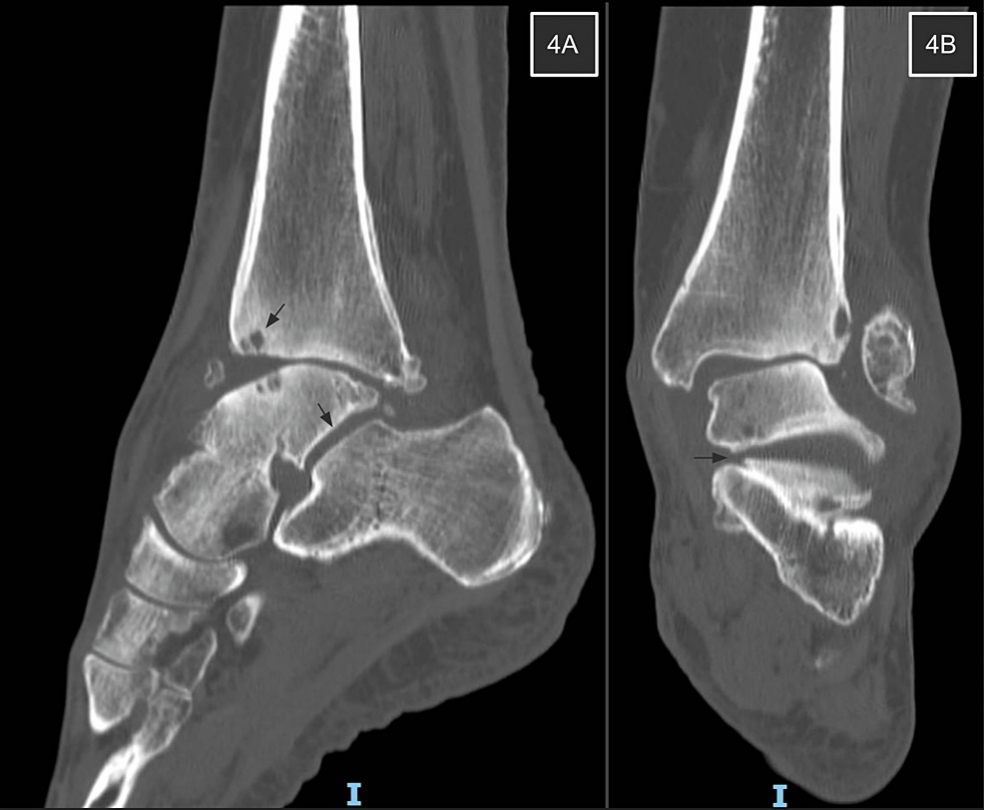
Ankle CT scan 16 months post-FVFG CT scans as shown in 4A and 4B reveal small periarticular cysts of the ankle joint and confirm the viability of the talus. Subtalar joint congruency is well preserved.

**Figure 5:**
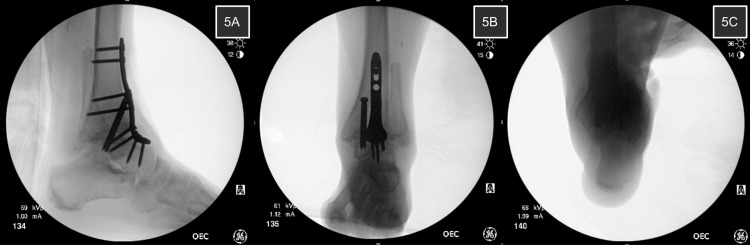
Intraoperative fluoroscopic images The final fixation construct is pictured in 5A and 5B demonstrating appropriate ankle joint positioning in the sagittal and coronal planes, respectively. 5C confirms a neutral hindfoot position with a simulated hindfoot alignment view.

Treatment for her VAD consisted of ankle arthrodesis. Her preoperative radiographs demonstrate end-stage tibiotalar joint osteoarthritis with valgus talar angulation and continued proximal migration of the lateral malleolus (Figures 3A-3C). A preoperative CT was also obtained (Figures 4A-4B). A standard anterior approach was utilized. Ankle joint preparation was performed with standard techniques. Deformity correction was performed by aggressive preparation of the medial tibiotalar joint with bone graft augmentation in the joint laterally. Due to the history of multiple nonunions, a multitude of osteobiologics were used in her arthrodesis to enhance her healing potential. Bone marrow aspirate concentrate was harvested from her iliac crest and combined with a biologic bone graft substitute, which was packed throughout the fusion site with an extra graft placed laterally where her joint had collapsed. The ankle joint was then positioned anatomically and fixated with an anterior plate and one 7.0 mm interfragmentary static screw. The hindfoot and forefoot positions were evaluated intraoperatively for residual deformity. The foot and ankle alignment were corrected well after ankle fusion, and no additional procedures were performed. Final fluoroscopic imaging is shown in Figures 5A-5C. A drain was placed postoperatively. The patient was started on a bone stimulator on postoperative day one. The patient was kept non-weight bearing for 12 weeks in a splint or cast. There were no complications postoperatively.

## Discussion

The occurrence of VAD following FVFG is a well-known donor site complication in skeletally immature patients. In contrast, adults with stable congruent ankle joints are not expected to suffer from this complication at all. This case report demonstrates the possibility of severe VAD following FVFG in an adult patient population with generalized joint hypermobility. Not unexpectedly, the syndesmotic ligament in adults with EDS may lack the robust nature required to support the ankle after the removal of the fibular shaft. Without the ligamentous support of the proximal fibula at the knee, all of the force that is transmitted through the fibula during ambulation is dispersed to just the distal remnant and syndesmotic ligaments, causing it to displace.

In adults with syndesmotic instability following trauma, syndesmotic stabilization in the form of screws or sutures is used across the distal tibiofibular joint to maintain stability and reduction while the ligaments heal. In children undergoing FVFG harvest, this is recommended on certain occasions when the risk of developing VAD is deemed the highest: younger age and short fibular remnant [9]. We think adults with EDS may benefit from syndesmotic stabilization or even primary syndesmotic fusion following FVFG due to the risk of syndesmotic instability.

Syndesmotic stabilization commonly consists of one or two quadricortical screws or flexible suture devices made of ultra-high molecular weight polyethylene. Flexible suture devices have the advantage of allowing some degree of anatomic motion of the tibiofibular joint to reproduce more anatomic ankle joint biomechanics during gait. On the other hand, quadricortical screws may be stronger against resisting valgus forces on the fibula than sutures. Studies have shown little harm in anatomically placed syndesmotic screws in adults in the trauma setting, with their main downside being their reduction in talar rotation and translation in the ankle joint [10]. Syndesmotic fusion has the advantage of adding even greater resistance to translational forces in cases where syndesmotic screws can fail due to high stress, with the disadvantage of creating more rigid and constricting ankle joint kinematics.

The potential benefits of syndesmotic stabilization or primary fusion, in this case, include the prevention of end-stage arthritis and the need for major surgery. Quality of life scores in patients with end-stage ankle arthritis have been reported to be as low as those of patients suffering from diseases such as depression, Parkinson's, end-stage kidney disease, and diabetes, exemplifying the significant morbidity of this condition [11].

The mainstay of treatment for end-stage ankle arthritis is ankle arthrodesis or ankle replacement. Due to this patient's absence of an anatomic lateral malleolus, she was not a candidate for ankle replacement. Ankle arthrodesis is effective at removing pain from arthritis but has the downside of affecting normal biomechanics during gait, increasing the wear of surrounding joints through dissipated stress.

We are not sure if this complication could have been avoided. Although we recommend consideration of syndesmotic stabilization or syndesmotic fusion in adults with EDS undergoing FVFG, it is possible that ankle arthritis would result in this patient population regardless. If the syndesmosis was eliminated through screw placement or fusion at the time of FVFG, there is still the possibility of anterior or posterior talar translation due to joint hypermobility, resulting in abnormal wear and eventual joint collapse. In light of this possibility, free fibular graft harvest may be a relative contraindication for patients with EDS. Harvesting of structural grafts from alternative donor sites that do not depend on ligamentous support, such as iliac crest, could be considered primarily in this subset of patients.

One potential drawback to this case report is the relatively short follow-up time and, thus, the inability to assess for full radiographic healing of the arthrodesis at the time of publishing. The patient was also not genetically tested for EDS but met clinical criteria, which represents the majority of patients with a working EDS diagnosis. In addition, based on a validation study of the Beighton score, her score is 99% specific for her diagnosis based on her age and sex [12].

## Conclusions

This case report details a significant complication of end-stage VAD in an adult with EDS following FVFG that resulted in ankle arthrodesis. It is hypothesized that the patient's ligamentous laxity enabled proximal migration of her fibula post-FVFG which led to the rapid development of VAD with end-stage arthritis. It is not known whether syndesmotic stabilization or fusion would prevent this complication, but we think it is low risk with potential benefit in this patient population. At a minimum, we recommend counseling patients with EDS on potential risks and close monitoring of these patients for donor site complications postoperatively. Alternatively, consider harvesting structural bone grafts from an alternative site in this patient population to avoid this complication.
